# USSTRIDE program is associated with competitive Black and Latino student applicants to medical school

**DOI:** 10.3402/meo.v19.24200

**Published:** 2014-05-23

**Authors:** Kendall M. Campbell, Thesla Berne-Anderson, Aihua Wang, Guy Dormeus, José E. Rodríguez

**Affiliations:** 1 Center for Underrepresented Minorities in Academic Medicine, College of Medicine, Florida State University, Tallahassee, FL, USA; 2 College and Pre-College Outreach, College of Medicine, Florida State University, Tallahassee, FL, USA

**Keywords:** minority health, medical education, pipeline, healthcare workforce, minorities in medicine, medical school admission

## Abstract

**Purpose:**

We compared MCAT scores, grade point averages (GPAs), and medical school acceptance rates of Black and Latino students in an outreach program called Undergraduate Science Students Together Reaching Instructional Diversity and Excellence (USSTRIDE) to non-USSTRIDE students. We hypothesized that Black and Latino participants in USSTRIDE had higher acceptance rates to medical school, higher MCAT scores, and college GPAs when compared to other Black and Latino medical school applicants from our institution.

**Methods:**

The academic performance (GPAs and MCAT scores) and acceptance and matriculation rate data on all Black and Latino Florida State University applicants to any medical school from 2008 to 2012 were collected from the AIS/AMCAS database and separated into two comparison groups (USSTRIDE vs. Non-USSTRIDE). Independent sample T-tests and chi-square analysis, Cohen's D test, and odds ratios were determined.

**Results:**

Average science GPA was 3.47 for USSTRIDE students (n=55) and 3.45 for non-USSTRIDE students (n=137, p=0.68, d=0.0652). Average cumulative GPA was 3.57 for USSTRIDE students and 3.54 for non-USSTRIDE students (p=0.45, d=0.121). Average MCAT score was 23 for USSTRIDE students and 25 for non-USSTRIDE students (p=0.02, d=0.378). Twenty-three percent of accepted USSTRIDE students and 29% of accepted non-USSTRIDE students had multiple acceptances (p=0.483, OR 1.38, 95% CI 0.52–3.88). Forty-nine percent of non-USSTRIDE students and 75% of USSTRIDE students matriculated in medical school (p=0.001, OR 3.13 95% CI 1.51–6.74). About 78.6% of USSTRIDE students matriculated at FSU's medical school compared to 36.2% of non-USSTRIDE students (p<0.01).

**Conclusions:**

USSTRIDE and non-USSTRIDE students had similar science and cumulative GPAs. USSTRIDE students' MCAT scores were lower but acceptance rates to medical school were higher. Participation in USSTRIDE is associated with increased acceptance rates for Black and Latino students to our medical school. This finding is true for other medical schools as USSTRIDE students are as likely as non-USSTRIDE students to have multiple acceptances.

Given the increasing diversity of the United States, the need for a more diverse physician pool is apparent. We know that minority faculties tend to care for minority patients (1) and that minority students need minority faculty mentors (2) to provide support and encouragement throughout their medical school experience. We also know that working to increase numbers of underrepresented minorities in medicine begins long before medical school as evidenced by pipeline programs designed to increase numbers of this group (3). Pipeline programs have been designed to provide educational and social support for high school and undergraduate students with the hope of increasing interest in science and math and eventually leading to careers in the health professions.

Although high school minority student pipeline programs are common (4–6), less has been published in the medical literature regarding college or university-level pipeline programs for medicine (7, 8). Many college and university programs are also focused on increasing medical college admission test (MCAT) scores and grade point averages (GPAs), as the means of increasing acceptance (3). Other professions, such as dentistry, have published numerous papers on this topic, especially the ‘pipeline’ programs for college and university undergraduates (9–12).

One such program, the Undergraduate Science Students Together Reaching Instructional Diversity and Excellence (USSTRIDE) program is progressing toward increasing the numbers of Latino and Black students accepted to medical school. USSTRIDE is part of the larger Science Students Together Reaching Instructional Diversity and Excellence (SSTRIDE) program that also features precollege and rural components, and was created by legislative mandate to address the shortage of rural and minority physicians in the state of Florida (13). USSTRIDE is a learning community that provides nurturing academic and social support services and mentoring to its student participants. To be admitted to USSTRIDE, students must have a successful interview with the college of medicine outreach and advising office, have a Scholastic Achievement Test (SAT) score of at least a 1,050/1,800, have a 3.0 or higher college GPA, complete an application for membership, and submit two letters of recommendation. Any precollege SSTRIDE student who has been in the outreach program prior to matriculation at the Florida State University is automatically admitted into USSTRIDE. USSTRIDE is open to students of all ethnicities and does not restrict its membership to those with a Black or Latino background. Because instructional diversity is one of the stated missions of the program, Black and Latino students are encouraged to apply. There are approximately 50 spaces available for new members each academic year. USSTRIDE students receive premedical advising, early clinical experiences, and public speaking tutorials and are placed in study groups for tutoring. They also receive mock interviews to simulate the actual medical school interview and leadership development training to create leaders in healthcare. Their personal statements are reviewed and they are offered membership and leadership opportunities within premedical organizations. Physician externship opportunities are provided, along with MCAT review sessions and mentorship with precollege and medical students. USSTRIDE students also receive close mentoring by faculty members. USSTRIDE is open to applications in the first 3 years of college, and many of the USSTRIDE students are members during their entire undergraduate career.

We sought to determine the effectiveness of the USSTRIDE program by comparing undergraduate Latino and Black students who participate in this program to undergraduate Latino and Black students who do not. Parameters explored included GPA and MCAT scores, as well as entrance to medical school. This study received institutional review board approval. We hypothesized that Black and Latino participants in USSTRIDE had higher acceptance rates to medical school, and higher MCAT scores and college GPAs, when compared to other Black and Latino medical school applicants from our institution. For the sake of simplicity, unless otherwise noted, all mentions of students in this manuscript mean Black and Latino students.

## Methods

This was a retrospective cohort study based on data collected from 2008 to 2012. This study received institutional review board approval by the human subjects committee. The academic performance (GPAs and MCAT scores) and acceptance and matriculation rate data on all Black and Latino Florida State University applicants to any medical school from 2008 to 2012 were collected from the AIS/AMCAS database and separated into two comparison groups (USSTRIDE vs. non-USSTRIDE). We included only those applicants with a GPA of 3.0 or higher, as per requirement for USSTRIDE membership. Independent sample *t*-tests were used to compare science GPA, general GPA, and MCAT scores between both groups. Chi-square testing was used to compare USSTRIDE and non-USSTRIDE students’ probability of getting into medical school. Cohen's *d* test was performed to calculate the effect size of MCAT and GPA comparisons, and odds ratios were calculated to compare matriculation and multiple acceptance rates. All statistics were run using SPSS version 21.

## Results

The AMCAS/AIS database had GPA and MCAT data for 137 non-USSTRIDE students and 55 USSTRIDE students, and it had multiple acceptance data for 40 USSTRIDE students and 70 non-USSTRIDE students. The average science GPA was 3.47 for USSTRIDE students (*n*=55) and 3.45 for non-USSTRIDE students, (*n*=137, *p*=0.68, *d*=0.0652). The average cumulative GPA was 3.57 for USSTRIDE students and 3.54 for non-USSTRIDE students (*p*=0.45, *d*=0.121). The average MCAT score was 23 for USSTRIDE students and 25 for non-USSTRIDE students (*p*=0.02, *d*=0.378). Forty-nine percent of non-USSTRIDE students matriculated in medical schools, and 75% of USSTRIDE students matriculated into medical schools (*p*=0.001, OR 3.13 95% CI 1.51–6.74). Twenty-three percent of accepted USSTRIDE students and 29% of accepted non-USSTRIDE had multiple acceptances (*p*=0.487, OR 1.38, 95% CI 0.52–3.88). Nearly 78.6% of USSTRIDE students who matriculated at the FSUCOM were compared to 36.2% of non-USSTRIDE students (p<0.01).

## Discussion

Our results suggest that USSTRIDE students are more likely to be accepted into medical school, despite lower MCAT scores and similar GPAs to those of underrepresented minorities who did not participate in SSTRIDE. Although we expected USSTRIDE students to do better than their non-USSTRIDE counterparts, we also expected that it would come with higher objective measures (GPA and MCAT). Other pipeline programs have been described in the literature, citing better preparation through improved GPA and MCAT as the principle reason for higher acceptances (14). Our study shows higher acceptance without improvement in grades or MCAT. These data suggest that the interventions provided by USSTRIDE aid in undergraduate Black and Latino student acceptance to medical school; and although there were more students with multiple acceptances in the non-USSTRIDE group, that difference was not found to be statistically significant. This finding suggests that USSTRIDE participation increases likelihood of matriculation in FSU's college of medicine. Forty-nine percent of non-USSTRIDE students were admitted to medical schools. This is close to the average acceptance rates for this group of students with MCATs and GPAs that fall into this range (52% of Hispanic applicants and 65% of Black applicants were admitted to medical school from 2010–2012) (15). USSTRIDE students in our sample, however, exceed the national average acceptances for their MCAT score (42.6% acceptance rate in 2009–2011) (16) again suggesting that USSTRIDE has a distinct benefit for this group of students.

The question of the contradictory findings of higher acceptance rates in the group with lower MCAT scores may not be as surprising as we originally thought. A recent study of the SAT/ACT use in college admissions shows that those tests are not predictors of college success as measured by GPA and graduation rates (17). However, it did show that high school GPA was a strong predictor of success. If applied to the MCAT, our study shows the same to be true for medical students: GPA matters more than MCAT. USSTRIDE students had a similar GPA to the non-USSTRIDE students, indicating that the more valuable predictor of success mattered more in the selection of medical students at our institution.

There are, however, significant limitations to our study. In comparing the two groups there are higher MCAT scores for the non-USSTRIDE students, contrasted with the higher medical school acceptance rate for USSTRIDE students. Because USSTRIDE selection happens years before students apply to medical school, there is no way to predict their MCAT scores. It is possible that our USSTRIDE selection committee may have a good eye for identifying Black and Latino students who will be successful in medical school admission. That could explain our findings were it not for the fact that the majority of USSTRIDE students were members of precollege SSTRIDE. There are currently no models available that can predict which Black and Latino high school students will have the academic firepower to be admitted to medical school. A study of the selection process of precollege SSTRIDE might elucidate this issue, and will help to determine what it is that makes the USSTRIDE student more successful in medical school admission.

Another limitation of our study was the small sample size of our population. One might ask why were there so few USSTRIDE Black and Latino students if 50 are accepted each year? Theoretically that should produce almost 200 Black and Latino USSTRIDE students. Although most USSTRIDE students apply to medical school, they are not limited to medical school applications. And although USSTRIDE is designed to be a disadvantaged student enrichment program, students are not excluded based upon race. There is a large rural component to USSTRIDE and those students are mostly white. For that reason, there is a relatively small cohort of USSTRIDE Black and Latino students.

Seventy-eight percent of matriculated USSTRIDE students matriculated at our college of medicine. With such a large percentage admitted to our own institution, this raises the concern for selection bias within our medical school admissions process. Our admissions process is not unlike other medical schools. Our admissions committee is made up of an interview committee and a selection committee. The school makes a concerted effort to screen all minority medical school applicants regardless of GPA or MCAT score. Once the screening process is complete, the dean of admissions decides whether the student will be interviewed. The student is then interviewed by two faculty members and is discussed at the weekly interview committee meeting. The interview committee makes recommendations to the selection committee; the latter committee ‘selects’ who is offered admission. The admissions committee uses a holistic approach to all applicants, and the MCAT is not the only factor in determining who will be interviewed.

The finding that 25% of USSTRIDE and 29% of non-USSTRIDE students had multiple acceptances shows that USSTRIDE students were as competitive as non-USSTRIDE students at other institutions, in spite of their significantly lower MCAT score. It is also plausible that USSTRIDE plays a role in FSU brand loyalty, e.g. they choose to come here because it was their undergraduate institution.

The Florida State University College of Medicine is a community-based medical school with the mission of educating and developing exemplary physicians who practice patient-centered healthcare, discover and advance knowledge, and are responsive to community needs, especially through service to elder, rural, minority, and underserved populations (18). We believe this intervention is increasing the numbers of underrepresented physicians who care for the underserved in higher percentages than other groups (19). Benefits that we note from the interventions provided to USSTRIDE students include an enhanced work ethic, higher rates of retention, improved critical thinking skills, improved communication skills, and improved understanding of themselves and others. Next steps include further analysis of the services provided by USSTRIDE in order to define more clearly how services are beneficial, and to quantify the benefits these services have for USSTRIDE students of all races and ethnicities. We are also working on increasing our data set to see whether we can draw more conclusions about USSTRIDE student USMLE step 1 scores as compared to non-USSTRIDE students.
